# Features of electron gas in InAs nanowires imposed by interplay between nanowire geometry, doping and surface states

**DOI:** 10.1038/s41598-017-03415-3

**Published:** 2017-06-13

**Authors:** V. E. Degtyarev, S. V. Khazanova, N. V. Demarina

**Affiliations:** 1 Physics Department, Nizhniy Novgorod State University, Gagarin Ave. 23, 603950 Nizhniy, Novgorod Russia; 2 Peter Grünberg Institute-2, Forschungszentrum Jülich, D-52425 Jülich, Germany and Jülich-Aachen Research Alliance-Fundamentals of Future Information Technology, Jülich, Germany

## Abstract

We present a study of electron gas properties in InAs nanowires determined by interaction between nanowire geometry, doping and surface states. The electron gas density and space distribution are calculated via self-consistent solution of coupled Schroedinger and Poisson equations in the nanowires with a hexagonal cross-section. We show that the density of surface states and the nanowire width define the spatial distribution of the electrons. Three configurations can be distinguished, namely the electrons are localized in the center of the wire, or they are arranged in a uniform tubular distribution, or finally in a tubular distribution with additional electron accumulation at the corners of the nanowire. The latter one is dominating for most experimentally obtained nanowires. N-type doping partly suppresses electron accumulation at the nanowire corners. The electron density calculated for both, various nanowire widths and different positions of the Fermi level at the nanowire surface, is compared with the experimental data for intrinsic InAs nanowires. Suitable agreement is obtained by assuming a Fermi level pinning at 60 to 100 meV above the conduction band edge, leading to a tubular electron distribution with accumulation along the corners of the nanowire.

## Introduction

Electron and hole gas in semiconductor nanowires of the sizes compared to the de Broglie wavelength is a fascinating object since its properties are determined by quantum-mechanical effects. Moreover, III/V semiconductors offer a wide range of opportunities to change the nanowire material composition, geometry and heterostructure design. This, in turn has a lot of implications, namely nanowires act as a test bed for detailed study of fundamental physics in low-dimensional systems as well as a perspective platform for numerous applications in nanoelectronics, optoelectronics, and spintronics^1–4^. The quantization in the potential landscape of a nanowire is ruled by both the Schroedinger and Poisson equations with appropriate boundary conditions at the surface. Thus, the state of a nanowire surface might strongly govern both charge carrier distribution and density within a nanowire. This apparently holds for InAs surface which is known for its high density of surface states in particular after exposure to air^5–13^. It is believed that due to the dominating donor-type surface states, supplying electrons to nanowire body, even intrinsic InAs nanowires have high n-type conductivity without additional doping^14–24^. In addition, low contact resistance of InAs nanowires^25^ is another indication for the formation of an electron accumulation layer at the nanowire surface. It is natural to expect that the electron gas formed in InAs nanowires due to the surface states would possess different features depending on the surface states and the nanowire diameter^14, 23, 26–33^. So far there exist several studies of electron and hole gas properties in core-shell nanowires, i.e. nanowires comprising a core and a shell composed, correspondingly of a narrow and wide band gap semiconductors, such as GaAs/AlGaAs^34^ and GaN/AlGaN^35^. There the charge carriers populate the core, thus, the influence of surface states has been excluded. Moreover, the impact of a nanowire width and crystal temperature has been neglected. We are going beyond the existing theoretical studies and focus on the formation and properties of an electron gas in intrinsic and n-doped hexagonal shaped InAs nanowires taking into account surface introducing the energy at which the Fermi level positioned at the surface as parameter. We show that the state of the surface as well as nanowire width and doping strongly define the type of electron space distribution which varies from an almost homogeneous to a strongly inhomogeneous one with a distinct electron accumulation along the corners of the hexagonal nanowires. We evaluate the ratio of electrons accumulated in the corners to those confined along the nanowire facets in dependence on the nanowire geometry as well as on nanowire doping. We calculate an electron density in intrinsic and n-doped InAs nanowires for different position of the Fermi level at the nanowire surface as well as a nanowire width and crystal temperature and present the long awaited comparison of obtained results with those for nanowires with a cylindrical cross-section as well as available experimental data.

## Theoretical model

**Figure 1:**
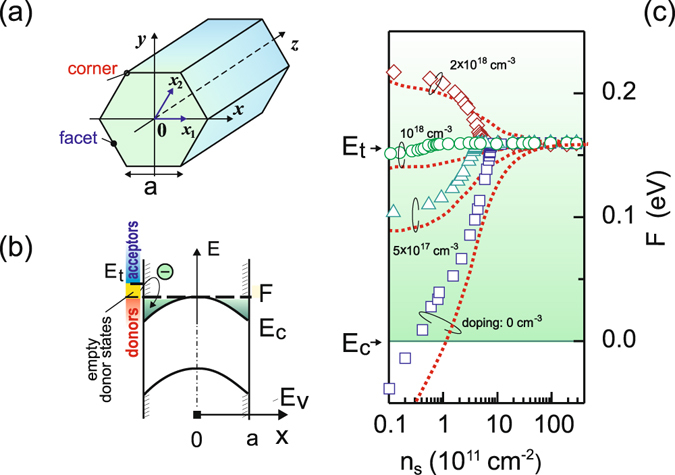
(**a**) Sketch of a nanowire. (**b**) Schematic band diagram of InAs nanowire with the donor- and acceptor-type surface states and electron accumulation region at the nanowire surface where *E*_c_ and *E*_v_ denote the conduction and valence band edge, correspondingly, and *F* is the Fermi level. (**c**) The Fermi level energy as a function of the total surface states density for the intrinsic and n-doped circular InAs nanowire (radius 50 nm) with the doping set to 5 × 10^17^ cm^−3^, 10^18^ cm^−3^, and 2 × 10^18^ cm^−3^ and the lattice temperature (*T*) of 4 K (symbols) and 300 K (dotted line).

In our theoretical study we consider InAs nanowires similar to those reported in ref. 36. In accordance with experimental findings^19, 23, 36^ we assume that the nanowire has a hexagonal cross section and a homogeneous crystal lattice with the zinc blende structure. We describe the cross section with a side length *a* (Fig. 1a) and assume an infinitive nanowire length. We consider both intrinsic and *n*-doped nanowires.

In order to describe in nanowires an electron gas distribution and a density, it is required to calculate the electronic band structure. Thus, we are solving the two-dimensional Schroedinger equation for the envelop function *Ψ*_α_ (*x*_1_, *x*_2_) of the quantum state α with the eigen energy *E*_α_ within the effective mass approximation

1 $$-\frac{{\hslash }^{2}}{2{m}^{\ast }}\sum _{i,j=1}^{2}\frac{\partial }{\partial {x}_{i}}(\frac{\partial }{\partial {x}_{j}}{{\rm{\Psi }}}_{\alpha }({x}_{1},{x}_{2}))-q\phi ({x}_{1},{x}_{2}){{\rm{\Psi }}}_{\alpha }({x}_{1},{x}_{2})={E}_{\alpha }{{\rm{\Psi }}}_{\alpha }({x}_{1},{x}_{2})$$

where m* is the electron effective mass (m* = 0.023 *m*_0_ in InAs), *m*_0_ is the free electron mass, *q* is the elementary charge. We assume that the envelop function vanishes at the surface of the nanowire. The electrostatic potential *φ* (*x*_1_, *x*_2_) is determined via solution of the two-dimensional Poisson equation

2 $$\sum _{i,j=1}^{2}\frac{{\rm{\partial }}}{{\rm{\partial }}{x}_{i}}(\frac{{\rm{\partial }}}{{\rm{\partial }}{x}_{j}}\phi ({x}_{1},{x}_{2}))=-\frac{\rho ({x}_{1},{x}_{2})}{\varepsilon {\varepsilon }_{0}}.$$

where *ε* is the dielectric permittivity (*ε* = 15.15 in InAs), *ε*_0_ is the vacuum permittivity and the electrostatic charge density *ρ* comprises the concentration of fully ionized donor atoms which are assumed homogeneously distributed over the nanowire body and the electron concentration *n*(*x*_1_, *x*_2_):

3 $$\rho ({x}_{1},{x}_{2})=q({N}_{d}^{+}({x}_{1},{x}_{2})-n({x}_{1},{x}_{2})).$$

Here *n*(*x*_1_, *x*_2_) is given by

4 $$n({x}_{1},{x}_{2})=\sqrt{\frac{2m\ast {k}_{B}T}{\pi {\hslash }^{2}}}\sum _{\alpha }{|{{\rm{\Psi }}}_{\alpha }({x}_{1},{x}_{2})|}^{2}{F}_{-1/2}(\frac{F-{E}_{\alpha }}{{k}_{B}T}),$$

where *F*_−1/2_ (…) is the Fermi-Dirac integral of order −1/2, *T* is the lattice temperature, *ħ* is Planks’ constant, *k*_B_ is Boltzmann’s constant, *F* is the energy of the Fermi level. We neglect a possible increase of ionization energy of dopants in nanowires due to dielectric confinement^37, 38^. We also neglect in the Schroedinger equation the electron-electron exchange-correlation potential which as it has been shown before^35^ has only minor influence on the calculated electron density.

The numerical solution of the coupled equations is performed using the finite-element method on the parallelogram-like mesh describing well the hexagonal shape of the nanowire cross section.

There exist several reports on surface states in InAs nanowires^14, 18, 21, 24, 32, 33, 39^. However, it has been rather common to use a model of the InAs surface which assumes that the neutrality level at the surface (*E*_t_) lies in the conduction band and the surface states located below it are donor- and above - acceptor-type (Fig. 1b)^5, 9^. The surface states density distribution in a single InAs nanowire has been so far revealed by fitting measurements on InAs field-effect transistor to a simulation resulting in a uniform density distribution as a function of energy in the range of 5 × 10^12^ to 1.5 × 10^13^ cm^−2^ ^14, 21, 24, 39^. The direct measurement of the surface states density of individual InAs nanowires detected an exponential distribution with a maximum of 10^13^ cm^−2^ eV^−1^ ^32, 33^.

The density of nanowire surface states as a function of energy might be also described by the *U*-type dependence, known for the surface of bulk InAs after oxidation at room temperature in air^5, 9–12^. As an example Fig. 1c shows the calculated position of the Fermi level with respect to the conduction band edge as a function of the total density of charged surface states *n*_s_^40^. The calculations have been performed for cylindrical InAs nanowire with *U*-type surface states distribution. However, we expect only minor changes of the presented tendency for nanowires with a hexagonal cross-section. In the intrinsic InAs nanowires the Fermi level is below the neutrality level, thus in accordance with the Fermi-Dirac function there is some probability that electrons are transferred from the surface states to the nanowire body. The increase in the surface states density leads to a shift of the Fermi level towards the neutrality level where it is pinned in both intrinsic and n-doped nanowires as soon as the surface states density exceeds 10^12^ cm^−2^. The latter is in agreement with the recent direct measurement of the Fermi level position at the surface of intrinsic InGaAs nanowires exposed to air^41^. What is more important, that Fig. 1 delivers a rather complete picture of the possible location of the Fermi level in dependence on various values of the density of surface states. For intrinsic nanowires (indicated in Fig. 1c as doping: 0 cm^−3^) the Fermi level might be shifted from the gap to the close vicinity of the neutrality level by increasing *n*_s_ from 10^10^ to 10^12^ cm^−2^. In n-doped nanowires the Fermi level lies not further than 0.1 eV below or above the neutrality level. Being armed with this knowledge, in the present theoretical study we describe the surface more generally, namely without introducing a specific type of the surface states energy distribution, we assign a certain position to the conduction band edge with respect to the Fermi level at the nanowire surface. As we showed above it can be related to the total density of surface states and for each model of the surface this relation might be rather different.

## Results and Discussion

**Figure 2:**
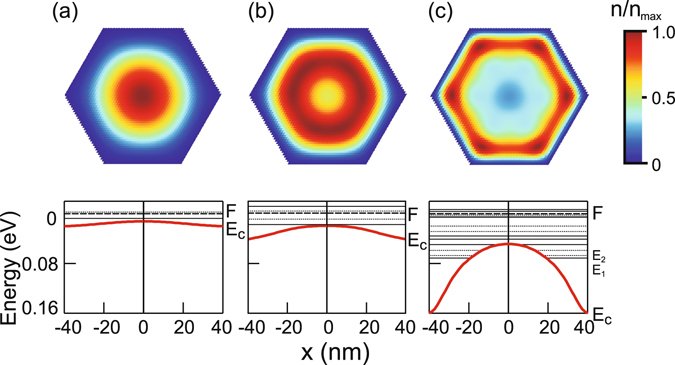
Upper: Two-dimensional electron distribution in a nanowire with a side length of 40 nm and the Fermi level positioned 0.02 (**a**), 0.04 (**b**), and 0.16 eV (**c**) above the conduction band edge at the surface (*T* = 4 K). Lower: The conduction band diagram and the eigen energy states (non-degenerate (solid line) and doubly degenerate (dashed line)) in the nanowire for the values of (*F* − *E*_c_) indicated above.

The surface state density determines the main features of electron distribution within the nanowire. Figure 2 (upper) shows an electron distribution for various positions of the Fermi level with respect to the neutrality level in the intrinsic nanowire. If the Fermi level is far from the neutrality level which corresponds to a low surface states density (Fig. 2a) the conduction band is almost flat (Fig. 2a (lower)) and electrons are confined in the centre of the nanowire with the density maximum located at the nanowire axis. The electric field introduced by the positive charge of the dominating donor-type surface states increases with an increase in surface states density and bends the conduction band downwards leading to the tubular like electron distribution at the surface (Fig. 2b). For even higher surface states densities when the Fermi level is pinned at the neutrality level, the tubular electron distribution is preserved while higher electron population forms at the nanowire corners in comparison with the facets (Fig. 2c).

**Figure 3:**
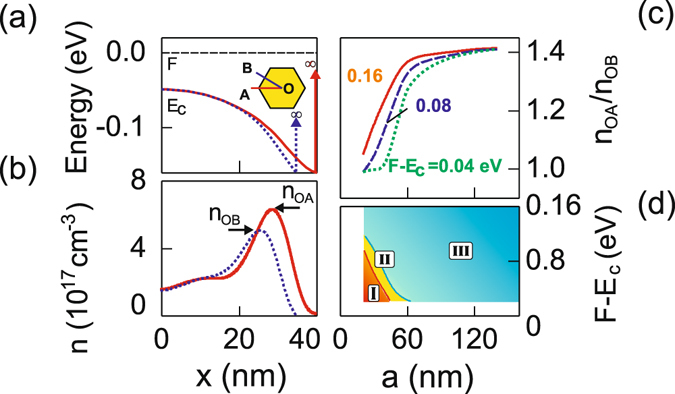
(**a**) The conduction band diagram plotted in the direction towards the nanowire corner (solid line) and the middle of the nanowire facet (dotted line) for the intrinsic InAs nanowire with *a* = 40 nm at *T* = 4 K. Inset: Sketch of a nanowire cross section with the indicated corner direction (OA) and facet direction (OB). (**b**) Three-dimensional electron density as a function of the coordinate plotted in the direction towards the nanowire corner (solid line) or middle of the nanowire facet (dotted line) for the intrinsic InAs nanowire with *a* = 40 nm at *T* = 4 K. (**c**) Ratio of the maximum electron density in the corner direction (*n*_OA_) to the maximum electron density in the facet direction (*n*_OB_) as a function of the nanowire side length *a* (*T* = 4 K). The Fermi level is positioned at the surface 0.04 (dotted line), 0.08 (dashed line), and 0.16 eV above the conduction band edge. (**d**) Two dimensional diagram of various electron distributions: (I) the centered distribution, (II) the tubular distribution, (III) the tubular distribution with the additional electron accumulation regions at the corners.

The origin of the inhomogeneous angular electron distribution has been so far explained by the shape of the wave-function of the lowest energy eigen states^35^ or interpreted by the repulsive Coulomb interaction between like particles requiring maximization of the interparticle distance^34^. We would like to give a more comprehensible interpretation of the effect. Figure 3a shows the position of the conduction band edge as a function of a coordinate in the direction towards the corner or the middle of the nanowire facet. The conduction band forms a triangular-like quantum well for electrons with the width determined by the side length of the nanowire and the depth dependent on the position of the Fermi level with respect to the conduction band edge thus the surface states density. Among these two potential wells considered independently, the well formed in the corner direction is wider than the quantum well formed towards the facet. Thus, the quantum energy states are lower in the corner well and energetically more favourable for electron population than those which form in the facet well. Consequently, the corner well will be more populated by electrons than the facet well. Apparently, in the nanowire there exists the full superposition of the wells with the width varying from the corner to the facet limiting case. However, the electron gas tends to form more densely in the vicinity of the more energetically preferable direction, thus at the corner of the nanowire which is shown in Fig. 3b.

Figure 3c shows the ratio of the electron maximum density towards the corner to the maximum density towards the middle of the facet as a function of the nanowire side length *a*. The ratio increases with increasing width *a* due to the raise of the absolute difference between the width of the corner and facet quantum wells. The same effect is observed for an increase in (*F* − *E*_c_) which gives rise to a stronger band bending, i.e. a more pronounced quantum well at the surface. Thus, electron accumulation regions are more easily formed in nanowires with a larger cross section and a higher surface states density (Fig. 3c). However, the difference between the electron densities at the corner and at the facet centre does not exceed 40% which implies that the neighbouring corner accumulation regions are always strongly coupled for basically any state of the surface. The calculation performed for room temperature (not shown) shows similar ratios of the maximum electron densities, thus raising the temperature has only little impact on the carrier distribution. We summarize the simulation results concerning the electron space distribution in Fig. 3d. One can see that the dominating type of the electron distribution is the tubular distribution with different strength of electron accumulation at the corners. The other two types of distributions develop only in relatively thin nanowires with a small amount of surface states.

**Figure 4:**
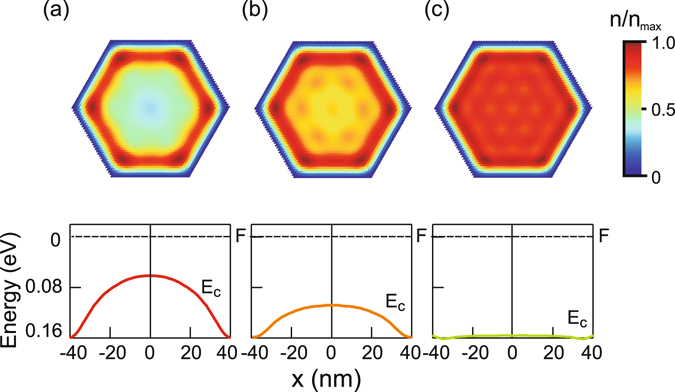
Upper: Two-dimensional electron distribution in a nanowire with a side length of 40 nm and the Fermi level positioned 0.16 eV above the conduction band edge at the surface; the n-type doping density is set to 5 × 10^17^ cm^−3^ (**a**), 10^18^ cm^−3^ (**b**), and 2 × 10^18^ cm^−3^ (**c**); *T* = 4 K. Lower: The conduction band diagram in the nanowire for the doping density indicated above.

The *n*-type doping of the nanowire introduces additional electrons into the nanowire which leads to flattening of the conduction band. Figure 4a–c shows that the increase of the donor density from 5 × 10^17^ cm^−3^ to 2 × 10^18^ cm^−3^ changes the quantum well shape from triangular to square-type which suppresses electron accumulation at the corners and the electrons almost homogeneously distribute within the cross-section (Fig. 4c, upper). This is opposite to the case reported before for core-shell structures^34, 35^ in which *n-*type doping increased the amount of accumulated electrons at the corners.

**Figure 5:**
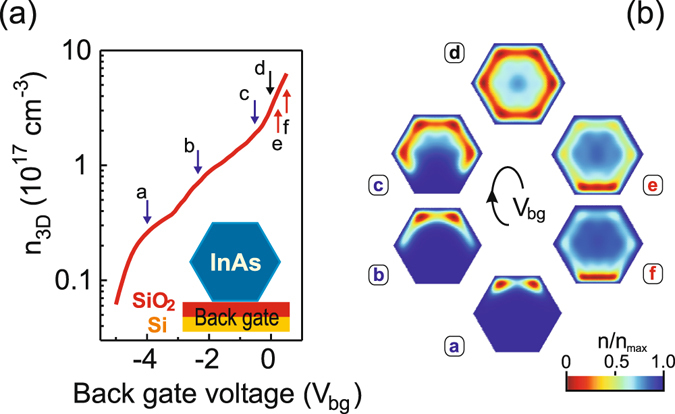
(**a**) Three-dimensional electron density as a function of the back-gate voltage applied to the InAs nanowire with the side length of 40 nm and the Fermi level positioned 0.16 eV above the conduction band edge at the surface at *T* = 4 K; the back-gate voltage is set to −4 V (a), −2.5 V (b), −0.5 V (c), 0 V (d), 0.1 V (e), 0.2 V (f). Inset: Sketch of a back-gated InAs nanowire. (**b**) Two-dimensional electron distribution in the InAs nanowire corresponding to the back-gate voltage indicated above.

The electron distribution might be also substantially ruled by applied gate voltage. In the case of a back gate the nanowire is positioned on a highly doped Si substrate and separated from it with a layer of Si oxide (Fig. 5, inset). We simulate the influence of the back gate via changing the boundary condition for the electrostatic potential in the Poisson equation on the facet laying on the dielectric by *V*_bg_, where *V*_bg_ denotes the back-gate voltage. Figure 5 shows the dependence of the electron density *n*_3D_ $({n}_{3D}={\iint }_{s}n({x}_{1},{x}_{2})d{x}_{1}d{x}_{2}/(3\sqrt{3}{a}^{2}/2))$ on the applied back gate voltage. The curve has a slightly step-like shape which is related to the corresponding behaviour of the area inside the nanowire depleted by the gate voltage. As it is shown in Fig. 5b the electric field of the negative applied gate voltage shifts electrons towards the upper part of the nanowire depleting the region in the vicinity of the gate area, thus leading to a decrease in the amount of electrons populating the nanowire. The electron density decreases faster when the boundary of the depleted region crosses the area with initially higher electron concentration. Similar to the results of ref. 34 the electron accumulation regions at negative applied back-gate voltage are preserved however they become slightly less pronounced. The positive gate voltage leads to formation of the strong electron accumulation region close to the back gate with a minor electron density within the rest of the nanowire cross section.

**Figure 6:**
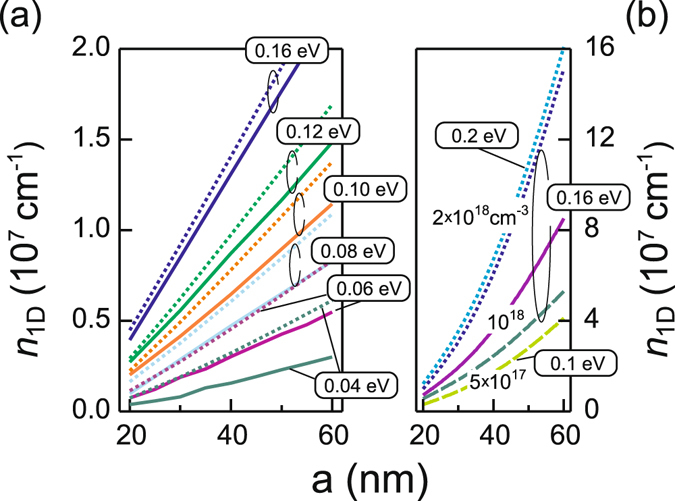
(**a**) One-dimensional electron density as a function of the nanowire side length plotted for various position of the Fermi level with respect to the conduction band edge at the surface for the lattice temperature *T* = 4 K (solid line) and 300 K (dotted line). (**b**) One-dimensional electron density as a function of the nanowire side length plotted for various position of the Fermi level with respect to the conduction band edge at the surface for the lattice temperature *T* = 4 K and n-type doping density set to 5 × 10^17^ cm^−3^ (dashed line), 10^18^ cm^−3^ (solid line), and 2 × 10^18^ cm^−3^ (dotted line).

In order to reveal the influence of the nanowire width on the electron population of the intrinsic nanowire we calculate the average one-dimensional electron density (${n}_{1D}=3\sqrt{3}{a}^{2}{n}_{3D}/2$) as a function of the nanowire side length for various position of the Fermi level with respect to the conduction band edge (Fig. 6a). The electron density increases with increasing side length. Obviously, an increase in the width of the quantum well lowers the energy of the quantized energy states which in turn increases the number of states positioned below the Fermi level which are populated by electrons. The linear behaviour of the dependence is determined by the required charge neutrality of the nanowire, which demands for an intrinsic nanowire that the total charge of the free electrons in the body of the nanowire is equal to the charge of the ionized donor-type surface states.

The one-dimensional electron density *n*_1D_ is given by *n*_1*D*_ ∝ *aN*_*sd*_, where *N*_sd_ denotes the two-dimensional density of charged donor-type surface states. Figure 6 displays also that lowering of the conduction band edge at the surface from 0 to 0.160 eV leads to the increase in the electron density due to the larger amount of electrons supplied by the donor-type surface states. The increase in lattice temperature affects the electron density only slightly for the Fermi level pinned at the surface (*F* − *E*_c_ = 0.16 eV) while when the Fermi level is far from the neutrality level (*F* − *E*_c_ < 0.060 eV) the electron density increases by more than 30% with the lattice temperature raise from 4 to 300 K. This we attribute to influence of the quantum well shape confining the electrons in the nanowire. Indeed, in the nanowire with the Fermi level far from the pinned position at the surface, only a very shallow triangular or even a flat potential is formed across the nanowire (Fig. 2a and b) with the energy states formed more densely than in the pronounced triangular-like quantum well which is typical for the pinned Fermi level (Fig. 2c). Obviously, at room temperature the Fermi-Dirac distribution broadens thus more energy states become occupied by electrons in case of the nanowires with unpinned than in the nanowires with the pinned Fermi level which yields the stronger absolute change of the electron density.

In order to reveal the correspondence between simulated and measured values of the electron density we compare the calculated electron density (Fig. 6a) with the experimental data presented earlier in ref. 23. The measurements have been performed by making use of the back-gate configuration thus, as shown in ref. 42 the values might be substantially overestimated. Following the conclusions of ref. 42 we correct the measured data assuming that the values are a factor of four larger than the according values from more precise Hall measurements. We also take into account that different environment conditions may substantially change the density of surface states, thus disengage the Fermi level at the surface. However, the experimental values are lower than those which would in accordance to the simulation correspond to the pinning position of the Fermi level at the surface of 0.16 eV above the conduction band edge.

We cannot attribute this straight forward to the presence of the additional acceptor-type states at the surface trapping free electrons, as the simulations (not shown) revealed that additional acceptor-type surface states with a density of up to 10^13^ cm^−2^ do not significantly decrease the free electron density, if a total density of surface states above 5 × 10^12^ cm^−2^ is assumed. However, the same simulation showed that for the density of surface states below 10^12^ cm^−2^ the additional acceptor-type surface states efficiently trap free electrons thus lowering the free electron density. The latter might imply that in the measured InAs nanowires the total surface states density has been below 10^12^ cm^−2^ and the Fermi level has not reached its settled position at the neutrality level and is positioned between 0.060 and 0.100 eV above the conduction band edge. According to Fig. 3d in these nanowires, an electron gas is formed having the tubular-like distribution which has pronounced electron accumulation regions as soon as the nanowire side length exceeds 40 nm.

The *n*-type doping modifies the dependence of the electron density on the width of the side facet from a linear to a parabolic one (Fig. 6b). The qualitative behaviour of the calculated curves can be again derived from the nanowire charge neutrality. Note, that the total charge of the nanowire comprises the charge of the donor and acceptor-type surface states with the density *N*_sd_ and *N*_sa_, correspondingly, ionized donor atoms due to the doping with a density *N*_d_^+^ and free electrons. Thus, the one dimensional electron density amounts to ${n}_{1D}\propto 2a({N}_{sd}-{N}_{sa})+{a}^{2}{N}_{d}^{+}$. For a relative low doping level of 5 × 10^17^ cm^−3^ the increase of the surface states density from a low to a high value, i.e. changing the Fermi level position at the surface from 0.1 to 0.16 eV above the conduction band edge in accordance with Fig. 1c, increases the electron density by 25%. For the high doping level with the density above 10^18^ cm^−3^ the surface states contribution to the free electron density is small and the electron density is mainly determined by the doping. Additional simulations (not shown) also reveal that an increase in lattice temperature affects the electron density only slightly. This we attribute partly to the assumption of the full ionization of the donor atoms used in our model.

**Figure 7:**
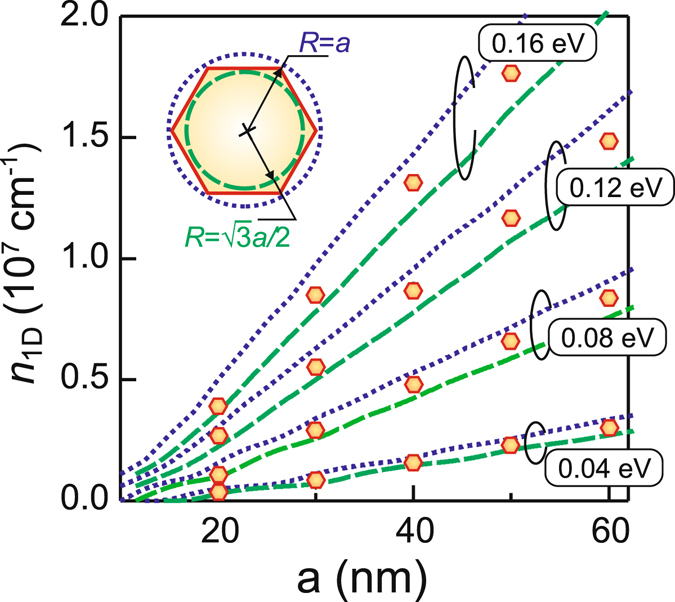
One-dimensional electron density calculated for the InAs nanowire with the hexagonal cross-section (symbols) and the circular cross section as a function of the nanowire side length plotted for the various position of the Fermi level with respect to the conduction band edge at the surface for the lattice temperature *T* = 4 K; the circular cross section corresponds to the circle circumscribed about (dotted line) and inscribed in (dashed line) the hexagon (inset).

The presented solution of the two-dimensional coupled Schroedinger and Poisson equations for nanowires with a hexagonal cross-section is substantially more time-consuming than that performed for cylindrical nanowires^40^. The assumption of the hexagonal cross-section leads to the eigen states different from those obtained following the simplified cylindrical approach and as a consequence more sophisticated type of electron gas space distribution. However, in some cases the problem is restricted to evaluation of an average electron density in a nanowire and does not require detailed analysis of electron gas features. Thus, the arising question is how close is the electron density value calculated for a cylindrical nanowire compared to the electron concentration obtained for corresponding realistic hexagonal nanowire. In order to answer this question we calculate a one-dimensional electron density in intrinsic InAs nanowire with both circular and hexagonal cross-section. We denote the circular cross-section radius *R* and consider two cases, namely *R* = *a* and $R=\sqrt{3}a/2$, which corresponds to the circle circumscribed about and inscribed in the hexagon (Fig. 7, inset). Figure 7 shows that for any state of the surface the one-dimensional density for the two limiting cases deviates from the “hexagonal” value by not more than 5%. This implies that first, the both limiting cases are acceptable for the simplified estimation of the electron density in a hexagonal-type nanowires and, second, the best agreement between two approaches will be reached for ${R}^{2}=3\sqrt{3}{a}^{2}/2\pi$, i.e. when the areas of the cross-sections are equal.

In the discussion part of the manuscript we address some questions which have not yet been raised. First, in our theoretical model we have so far neglected the effect of Rashba-type spin-orbit coupling on the electron energy states. Presence of this type coupling in nanowires has lately become particularly important as the recent experimental and theoretical studies^43–46^ showed that InAs nanowires can be successfully used for spin manipulation. This is possible that the strong electric field formed in InAs nanowires with the electron accumulation region lifts spin-degeneracy of the states which in turn might modify the discussed above results. Despite the main part of the simulation in this study has been performed for nanowires with hexagonal cross-section we restrict our description of this effect to a nanowire with a circular cross-section. This is possible, as we showed before, due to the similarity of the results obtained by using the both models. Following ref. 45 the Rashba term in polar coordinates is determined by the radial component of the electric field formed due to the positive surface charge and depends on the distance from the nanowire centre. Our calculations for InAs nanowires with a radius ranging from 30 till 100 nm show that the electric field formed due to the presence of surface states reaches its maximum value about 80 kV/cm close to the nanowire surface decreasing towards the nanowire centre. This is in agreement with the previous theoretical studies^45, 46^. This field causes corresponding shift of the energy states which we calculated for the zero electron wave-vector and it occurs to be in the range from 0.1 to 3 meV for the energy states which contribute their electrons into the nanowire electron density. However, we have found out that this energy spectrum evolution has a minor effect on the electron space distribution and electron density at low and room temperature. Thus, inclusion of the Rashba term into the Hamiltonian for our simulation should not change the conclusions drawn by our study. Second, in our study we consider InAs nanowires with a zinc blende crystal structure. This determines the electron effective mass value as well as a possible range of the Fermi level position at the nanowire surface. In InAs nanowires with a wurtzite crystal structure the electron effective mass is larger, and the Fermi level pinning occurs at about 0.2 eV above the conduction band edge (ref. 41) opposite to 0.16 eV, as we considered. We do not expect any qualitative change of the results for the wurtzite-type InAs nanowires. However, some quantitative modification of calculated dependencies like, for instance, ratio of the maximum electron density in the corner direction to the maximum electron density in the facet direction (Fig. 3(c)) or the electron density vs nanowire side length (Fig. 6) might occur.

## Conclusions

Our theoretical study based on the solution of coupled Schroedinger and Poisson equations for the intrinsic and *n*-type InAs nanowires with the hexagonal cross-section shows that the electron space distribution and the electron density in the intrinsic InAs nanowires are strongly affected by the charged surface states, nanowire width and doping. The dominating type of the electron space distribution is tubular with additional electron accumulation regions at the nanowire corners coupled to each other. The depletion of the nanowire with the applied back-gate voltage does not destroy the accumulation regions however might slightly suppress them. The introduction of the *n*-doping does affect the electron distribution for the doping level above 10^18^ cm^−3^ smoothing its inhomogeneity.
